# Physiology of the Circulation

**Published:** 1823-06-01

**Authors:** 


### PHYSIOLOGY OF THE CIRCULATION.

1. *On the Principles of Inflammation and Fever.* By C. E. LUCAS, M. D. Octavo, pp. 304. London, 1822.

"Causæ proximæ investigatio ad cognitionem morbi ducit amplissimam."—CELSUS.

2. *An Experimental Inquiry into the Nature, Cause, and Varieties of the Arterial Pulse; and into certain other Properties of the larger Arteries, in Animals with Warm Blood; illustrated by Engravings.* By CALEB HILLIER PARRY, M. D. F. R. S.

WE believe with Celsus, as expressed in Dr. Lucas's motto, that the *investigation* of proximate causes increases our knowledge of the nature of diseases, even although we may never discover those proximate causes themselves—in the same way as the search after a supposed hidden treasure led to riches, though the treasure itself was merely imaginary. On this account we should be sorry to find the inquiries respecting inflammation and fever abandoned, because we have not yet discovered clearly their proximate causes, or rather their incipient morbid phenomena. A great deal of light has been thrown on the nature and treatment of these diseases during the last twenty years; and much curious (we hope ultimately useful) physiological knowledge has been elicited in the same period, both in this and in other countries. Facts when once ascertained, and experiments when once made, are no longer the property of individuals but of the republic of science at large:—they may even be legitimately turned against the doctrines they were first called in to support. Indeed it is hardly in the nature of things that the surest conclusions can be drawn by the first observers of facts or projectors of experiments. It requires the conflicting opinions, arguments, and views of various individuals to adjust the value of original data, and keep them at their proper level in the scale of real knowledge afterwards. We are not sorry, therefore, to see authors, whose pretensions to originality are very limited, taking a careful and comprehensive review of the facts already in our possession, since it is probable that much valuable information is sometimes lost from the want of a proper application of it in this way. It is to remedy this last defect that Dr. Lucas has issued his volume into the world, " trusting that the consideration of facts bearing upon subjects so deeply connected with the science of medicine, will not prove wholly uninteresting "

It is evident that in an investigation of the principles of inflammation and fever, the state of the nerves and blood-vessels, healthy and morbid, must form the greater part of the inquiry. Dr. Lucas accordingly sets out with some physiological observations on the nervous system. Like Dr. Philip, he limits the *nervous power* to that resident in, and exercised by, the nerves, (though probably derived from the brain and ganglia) whilst the power exercised by the brain and spinal marrow conjointly, is designated *sensorial.* By the *former*, of course, the functions of conveying impressions to and from the sensorium, and thereby giving birth to sensation, motion, secretion, &c. are performed ;—while the sensorial power, as far as relates to the functions of animal life, is limited to the receiving impressions from, and communicating them to, the nervous power, comprising therefore the power of volition. The opinion of Dr. Philip that the power of motion is inherent in the muscular fibre, and distinct from the nervous, which last is only a stimulus calling the former into action, is not adopted by Dr. Lucas. " This (says he) is only interposing another and unnecessary link in the chain of causation, as not being proved by his experiments."

" Thus he (Dr. Philip) infers that the heart and blood-vessels are independent of the brain and spinal marrow, acting by their own inherent muscular power, because experiments prove that their functions may be continued for a certain time after the removal of those organs. But this is readily understood, when we recollect that they more immediately depend upon the ganglionic system, in itself forming, in the words of Dr. Philip, ' a secondary centre of nervous influence.' " P. 4.

The other experiment of Dr. Philip, by which it appeared that the excitability of muscles, whose nerves are entire, is sooner exhausted by the application of salt to their naked fibres, than when all communication with the sensorium is cut off by the division of their nerves, from which Dr. P.

concludes, that "the nervous influence, so far from bestowing excitability on muscles, exhausts it like other stimuli," our author also endeavours to invalidate.

"The obvious explanation, then, of the experiment in question, is, that the power of muscles, subjected to the double exhaustion of the agency of the will and the stimulus of the salt, is sooner exhausted than that of others subjected to the latter only from the power of volition having been withdrawn by the division of their nerves." 5.

We may also, says Dr. Lucas, suppose that muscles more readily obey, and are therefore sooner exhausted by their own natural stimulus, than by an unnatural one.

"Although, therefore, these experiments certainly prove that the muscles act by a power accumulated within themselves, yet I see no reason why this should be any other than the nervous power, especially as it is proved by other experiments, that the power both of the voluntary muscles and of the heart and blood-vessels 'may be influenced by agents acting through the nervous system,' even to its total extinction. There does not seem, therefore, from these experiments, any sufficient reason for the admission of the muscular power as distinct from that exercised by the nerves." 6.

Among numerous just, but not novel observations on the nervous system, Dr. Lucas deplores our ignorance of those lesions of the brain and nerves, or sensorial and nervous powers, which often destroy life without leaving cognizable traces of disorganization. In such cases we have only the evidence of symptoms during life, and the experience of the juvantia and ledentia, which, by the way, were all the aids possessed by the fathers of medicine, who could know little or nothing of morbid anatomy, and yet practised with considerable success. It happens, however, that in most diseases, even where the nervous system is primarily and exclusively affected, that the vascular system is soon drawn into morbid action; and here it is that pathological anatomy comes into our assistance.

The second chapter of Dr. Lucas's work is on "the action of the blood vessels;" and as it is necessary that we should have as clear an idea as possible of the state of the sanguiferous system in *health*, we shall here give some account of the second work at the head of this article, not only to make its valuable contents diffusively known, but to correct a host of misapprehensions and *misrepresentations* which have gone abroad respecting the opinions of that excellent physician, (Dr. Parry,) on the subject of the circulation. We too have been misconstrued, to say the least of it, and therefore we shall endeavour, in this article, to lay before our junior brethren as fair a statement of this intricate subject as our limited powers will permit.

Dr. Parry's work opens with a synthetical view of the experiments on which the subsequent deductions of the essay are founded. The experiments were made on different animals, and on different arteries of animals. They all tended to prove one leading fact, that arteries suffered no dilatation or contraction during the systole or diastole of the heart. We shall introduce one of the experiments as an example.

*September* 22, 1814. "In a ram, both carotid arteries were laid bare by Mr. George Norman, in the presence of Mr. Coombs, two others of his pupils, and myself.

"Notwithstanding the animal was greatly agitated, there was not in either carotid the least appearance of dilatation during the systole of the ventricle, or contraction during its diastole: nor, except when the animal breathed, was there any degree of loco-motion perceived in these arteries, which remained completely quiet and at rest. Nevertheless, when either artery was compressed between the finger and thumb, the pulse was very strong and distinct.

"Both arteries were adequately tied by Mr. Norman, each with a single ligature; after which, the ram stood and walked about well, apparently suffering little inconvenience." P. 2.

A great variety of experiments are afterwards detailed; but all tending to establish the fact, that no actual dilatation or contraction takes place in the arterial system from the action of the heart.*

We shall pass over the chapters on the structure, and on the power of arteries very cursorily. Dr. Parry's reasonings and experiments on the relative proportions and power of tonicity and elasticity in arteries, are, as usual, acute and accurate. They lead to conclusions very different from those of preceding physiologists, and more especially of Mr. Hunter. That gentleman considers—

"The natural pervious state of an artery, to be that to which the

---

* We may just state one here, where, from the size of the animal and of the vessel, there could be no source of fallacy. There were present Mr. Norman, Dr. Charles Parry, Mr. Coombs, Mr. Sewel, and others. Several inches of the right carotid artery of a large old horse were laid bare. The pulse was 48 in the minute. The artery was long and attentively examined. "No person could discover the least degree of dilatation or contraction of the artery during the systole and diastole of the heart. No motion of any kind occurred from respiration. In reality the artery was altogether quiescent." The vessel was examined with a strong lens, and with threads slightly wound round it. "But we could not discover any change of place, whether absolute or relative in these threads." P. 20. Numerous experiments of this kind made in the presence of many witnesses, must surely put the question at rest, respecting the visible or appreciable dilatation and contraction of arteries corresponding with the systole and diastole of the heart.

elastic power naturally brings a vessel, which has been stretched beyond, or contracted within, the extent which it held in a state of rest. The stretched is that state produced by the impulse of the blood in consequence of the contraction of the heart; from which it is again brought back to the natural state by the elastic power, perhaps assisted by the muscular." *On the Blood*, p. 116.

From this and some other passages, Mr. Hunter appears to think that the natural state of an artery during life is that to which the elastic power spontaneously brings it, when no longer forcibly dilated by the systole of the heart. But Dr. Parry's experiments tend to prove, on the contrary, "that during health the larger arteries of a living animal, as well under the diastole as the systole of the left ventricle, are in a state of distention, to which they are forcibly impelled by their contained blood, against their mechanical power of elasticity." 70.

In order to elucidate this point, Dr. Parry caused two ligatures, an inch and a half distant, to be made at the same instant on the carotid of a living sheep. The artery, measured half way between the ligatures, was $\frac{2}{4}$ of an inch in circumference. The blood being suffered to flow out of this included part, from a lancet puncture, the artery, in the same spot as before, measured in circumference only $\frac{160}{400}$ of an inch. Hence the circumference of the artery was immediately reduced $\frac{40}{400}$ of an inch by the mere evacuation of the blood contained in the artery. In a horse, the circumference of the right carotid artery was $\frac{316}{400}$ of an inch. It was firmly tied at some distance before the part measured, and a second ligature was applied an inch beyond the first. In an hour, the intermediate portion was punctured. The blood having coagulated in the artery, little more than serum flowed out. The vessel being now measured in the same place as before, was found to be only $\frac{252}{400}$ of an inch in circumference. In sixteen hours after the death of the animal by bleeding from the left carotid, the circumference of the artery at the same point as before, was $\frac{379}{400}$ of an inch. Hence, the *elasticity* was as $\frac{137}{400}$ of the circumference of the artery, and the *tonicity* as $\frac{127}{400}$.

"From these two last experiments, says Dr. P. it appears, that the forced distention of the artery does not necessarily depend on the impulse of the blood from the systole of the ventricle, since it existed when the communication with the stream of blood from the heart was interrupted by a ligature.

"As during the common circumstances of health, arteries are naturally distended beyond that degree, to which they would spontaneously contract by their elastic power, so they are capable of suffering a still greater degree of distention, from increased fulness of blood in the general system, or from those excessive determinations of blood to particular parts, which occur, on various occasions, in the animal frame. Thus it is generally admitted, that, if the passage of the blood through one arterial trunk is intercepted, the collateral branches, supplying the part, undergo an increased distention, in order fully or partly to compensate the loss.

"This conclusion was rendered very probable by what occurred in the ram, Experiment 15; in which one carotid had been tied nearly eleven months before, and the other was found much larger than any that we had before observed.

"In order, however, more accurately to investigate this point, the right carotid of a small ewe having been found to be in circumference $\frac{233}{400}$ of an inch, the left carotid was tied; a few minutes after which, the right had gained in circumference $\frac{24}{400}$ of an inch.

"In another ewe, the right carotid being $\frac{222}{400}$ of an inch in circumference, was again measured twelve minutes after the left had been tied, and was found to have gained in circumserence $\frac{98}{400}$ of an inch.

"I know of no actual measurements to prove that a similar effect takes place, in inflammation and other maladies, in the larger arteries supplying the parts so affected; but we may reasonably presume its existence from the tangible, and sometimes visible, increase of size in the artery, as well as from the great perceptible enlargement of the veins leading from the diseased parts.

"From various preceding considerations, it is natural, *à priori*, to conclude, that, when under the state not only of the usual healthy dilatation of arteries, but of that increased dilatation which often accompanies disease, the distending cause, which is a certain quantity or momentum of blood, is diminished, the elasticity will tend to contract them, so as, within certain limits, to accommodate them to the quantity of blood which they ought to convey. If, however, the elasticity should be inadequate to the requisite force or degree of contraction, the tonicity, or vital power, may assume the office of contraction where it was left by the elasticity, and carry it to the necessary extent.

"In order to ascertain how far this accommodation of arteries to the loss of blood from the system would reach, I suggested the Experiment 24; in which, the circumference of the right carotid in a ewe having been first raised, by a ligature on the left, from $\frac{222}{400}$ of an inch, to $\frac{320}{400}$, was successively diminished to $\frac{228}{400}$, $\frac{250}{400}$, $\frac{238}{400}$, $\frac{235}{400}$, $\frac{222}{400}$, $\frac{201}{400}$, $\frac{191}{400}$, and $\frac{161}{400}$ of an inch, by as many blood-lettings of eight ounces each, from the left jugular vein, till the death of the animal.

"So also in Experiment 27, the right carotid of another ewe, which was $\frac{222}{400}$ of an inch in circumference, was successively reduced to $\frac{196}{400}$, $\frac{178}{400}$, $\frac{170}{400}$, $\frac{145}{400}$, and $\frac{127}{400}$ of an inch, by similar bleedings from the same vein.

"Hence we may understand, how readily the arteries may become more or less capacious, in proportion to the quantities of blood which may either exist in the whole system, or may be determined to particular parts."

Dr. Parry thinks it probable, however, that the relative capacities of entire arteries, or certain parts of them, may be affected by other agents than the blood contained in them; as, for instance, from the mere exposure of arteries, some examples of which are given. From these and other experiments, it appears that the fibres of the middle coats of arteries have no power of elongation, like the radiated fibres of the iris, but merely of contraction, and that in dilatation they are passive. In respect to elasticity, it is obvious, that as arteries are already dilated beyond the degree which that agent, if not counteracted, would have permitted, any increase of dilatation must arise, not from an augmentation, but a diminution of the action of that power; hence we may conclude, that the cause of the common dilatation of the larger arteries, and still more of that which is preternatural, is the mechanical distension of the blood. 78.

### The Nature and Cause of the Arterial Pulse.

Physiologists are not yet agreed respecting the cause of the pulse. Haller plainly attributes it to the alternate dilatation and contraction of the artery from the impulse of the wave of blood,* corresponding with the systole and diastole of the left ventricle of the heart. "D'abord je me suis assuré que le sang, poussé par le cœur, dilate les arteres, et forme ce battement, qu'on appelle le pouls."—*Memoires sur le mouvement du Sang*. 33. He acknowledges, however, that frequently in living animals examined by dissection, no pulse, nor any dilatation of the artery, can be seen.

Bichat, finding that in denuded arteries there was no dilatation during the systole of the left ventricle, considered the pulse as chiefly owing to a kind of locomotion in the whole artery, wherein it springs against the finger in the abovementioned state of the ventricle.

"To illustrate the term *locomotion*, says Dr. P. let us suppose a piece of flexible cane, which we may move up and down in the direction of its length—may cause to advance or recede—may bend in various ways, or may even turn on its own axis, while at the same time, its diameter or size is in no respect changed." 92.

---

* This *wave* of blood along the arteries is perfectly incompatible, as we shall presently shew, with the fact that the contraction of the ventricle and sensation of the pulse are strictly *simultaneous* in all parts of the body.—*Rev.*

Dumas maintains, with Galen, that the dilatation and contraction of arteries are inherent in themselves, and independent of the mechanical impulse from the heart.

Richerand, with the greater number of preceding physiologists, attributes the pulse to an increase of diameter in the artery from ventricular impulse of blood behind. Portal is of the same opinion. Sœmmering contends for a similar explanation of the pulse, and the writers of our own country are almost universally agreed that the dilatation and contraction of the arteries depend on the systole and diastole of the left ventricle.

The question is not purely of philosophical curiosity, since it involves a point of consequence in the practice of modern surgery. Were a young surgeon taught to expect that he should be able to distinguish, by dilatation and contraction, an artery which he was required to lay bare, and find this test to be wholly wanting, his embarrassment might be considerable; and were a more experienced operator about to tie a deep-seated artery, and find himself unable to discover that artery by the touch, or having made his ligature, remain ignorant whether it was rightly applied till assured by the event, his anxiety may be readily conceived.

" In the course of our experiments," says Dr. P. " in which a carotid, separated from all its attachments, was fully exposed to view, and yet no movement was observable in it, we were sometimes equally surprized to find, that, when the finger was pressed against it, or even when it was removed out of its place by the force of the finger beneath it, or on one side, it was equally void of all pulse to the touch.

" A frequent repetition of the same results led to a discovery of what appears to be the nature of the pulse itself.

" The blood, in every part of the arterial system, from the mitral valves, outwards through the whole frame, to the right auricle, may be considered as a set of continuous columns, possessing little compressibility, and filling the tubes in which they are contained.

" When, by the contraction of the left ventricle, the blood included in it is forcibly expelled into the aorta, all these columns receive the shock of propulsion at the same instant. But the velocity during this systole being greater than during the diastole, the momentum, and consequently the impulse, in every direction, is also greatest in the systole. When, therefore, an artery is compressed with the fingers, in the usual mode of feeling the pulse, the blood, in consequence of the systole rushing into the artery with an increase of momentum, gives a stronger impulse of dilatation to the fingers, than from the less momentum which exists during the diastole, and thus produces the phænomenon of pulse.

"Hence, it appears, that the pulse is the effect, not of an extension of an artery beyond its usual diameter, but of a stronger effort, during the systole of the ventricle, than during its diastole, to restore the usual diameter of the artery, which had been diminished by compression.

"Since, also, the excess of velocity from the systole extends throughout the whole of the space compressed by the fingers, it is evident that the distending effort, producing the sense of pulsation, must also be felt throughout that space.

"Before this explanation could be fully admitted, it was necessary to ascertain the cause of the converse of this state; or the reason why a pulse was sometimes wanting in an artery exposed to view; and insusceptible of any mode of examination by the touch. Reiterated trials demonstrated, that this deficiency was owing to the following circumstances. The coats of the carotids are so firm, that when either impelled against any soft substance, or simply moved out of their place, these arteries readily recede; suffering no reduction of diameter, and, therefore, giving no sensation of a pulse. But if they are confined by any hard substance placed behind them, so as to resist a change of position from the pressure of the finger, or if they are squeezed between the finger and thumb, so as, in either case, to suffer a certain reduction of diameter, then the pulse never fails to exhibit itself.

"These latter experiments, therefore, while they show that the pulse does not exist under the mere contact of the finger with the artery, and, therefore, completely refute the supposed dilatation of an artery by the systole of the ventricle, as an object of touch; by ascertaining the precise circumstances under which a pulse does or does not exist, demonstrate, beyond all reasonable objection, the nature of that phænomenon.

"Hence we see the probable reason, why no pulse is said to have been discovered in those surgical operations, to which I have before alluded. If, however, the principle as to both states be well established, we have only sufficiently to diminish with the finger the diameter of an artery; and then, if it be of a certain size, and the course of blood through it, from the heart, be uninterrupted, we shall not fail to feel the pulse in that artery."

From these facts, Dr. Parry explains a phænomenon which was a stumbling block in the way of Mr. Hunter and other physiologists; viz. why a *denuded* artery exhibits no pulsation, while the latter is visible when the artery is surrounded with its natural coverings.

In order to discover this cause, says he, lay bare an artery and no dilatation is perceptible: but place behind it any hard substance that prevents its recession, and then the finger placed opposite to this on the artery, immediately feels a propulsive stroke at each systole of the left ventricle. In short, the whole which is necessary is, that the artery should have its diameter in a certain degree reduced; and then the substance reducing it, if not too ponderous, will be driven off in leaps, always according with the systoles of the heart, and often perceptible to the eye.

"In this way the pulsation of arteries may be frequently seen, when they are unusually compressed by muscles, or other intervening substances. Hence fæces in the colon; or other indurations in the abdomen, near, or in close contact with the aorta, by compressing that vessel, often both to the eye and touch, simulate aneurisms; and hence, aneurisms themselves, forming for the blood channels which are preternaturally compressed in various directions, suffer jerks from the impulse of the ventricle, which often shake all the neighbouring parts.

"It is not then the mere intervention between the eye or finger and an artery of any substances, whether hard or soft, that, conformably to the opinion of Mr. Hunter, make its pulsations more sensible; but it is because the substances thus intervening, often interrupt the current of blood through the artery, and therefore receive a shock, which may be tangible or visible, from each systole of the ventricle." 118.

In a former number of this Journal we expressed our conviction that the heart possessed the power of *expanding* its cavities, as well as of *contracting* them; and, consequently, that a suction influence was exerted on the venous system. It appears that Dr. Parry is of a similar opinion.

"Those," says he, "who maintain that the pulse is owing to the alternate dilatation and contraction of arteries, from the systole and diastole of the left ventricle, have probably deduced their conclusion from the analogy of the action of the heart itself. In this organ, they presume that each cavity is dilated in consequence of the distending power of the blood impelled into it, and then contracts itself in consequence of that distension. To me, however, it appears that this theory, however specious, is absolutely erroneous. The different compartments of the heart are so far from expanding, in consequence of the blood which is driven into them, that, *when altogether empty of blood, and even separated from the animal, they expand in a greater degree than when in their natural situation during life and healthy circulation.* Hence, Mr. Hunter himself, notwithstanding the view which he gives of the action of the heart, acknowledges that this organ, after death, has a larger volume than while the animal is living." 122.

Now if the chambers of the heart are capable of dilating themselves, they must as inevitably *suck* the blood from the venous system, as the expansion of a pair of single bellows draws in air through the pipe. There does not, therefore, appear any necessity for the *resiliency* of the lungs, either in the fœtal or subsequent state, though in the latter it is not impossible that it may have some operative effect.

In Dr. Parry's remarks on anomalies of the pulse, some curious instances are related, shewing the accuracy of attention which our author must have long been in the habit of bestowing on every form and phænomenon of disease.

"Twenty instances," says Dr. P. "have occurred to me of the total loss of pulse in the radial arteries, from various maladies of the *alimentary canal*, whether a considerable time before death, or under circumstances that admitted of recovery; while at the same time the pulse in the carotids was full and strong. In one instance of general dropsy, the patient had no pulse in either wrist for seventeen days; yet was restored to perfect health. Sometimes, before death, the pulse has been wanting in one radial only.

"I have seen a total loss of pulse in one arm, with coldness, but complete power of motion in that part; while the other arm was warm, and possessed a perfectly good pulse, but had lost all power of voluntary motion. These symptoms commenced suddenly, two or three days after parturition. The patient soon died, but a dissection was not obtained.

"In another case, a young man, labouring under pulmonary hectic, was found to have lost the pulse in one wrist, immediately after coming out of a warm bath. Several months afterwards, it had returned, though in an almost imperceptible degree.

"Another patient, a female of middle age, the mother of several children, affected with severe cough, was, apparently, in a state of convalescence, and walking about her house; when it was discovered that the pulse in one arm was wholly wanting. A few days afterwards she died suddenly. The whole course of the aorta was carefully examined; but no deviation from the healthy state could be perceived in it." 140.

Who would suppose that, after all Dr. Parry has written respecting the elasticity, tonicity, and ever varying diameters of arteries in the living system, he, and those who entertained similar opinions, should be represented as invariably considering arteries to be "*inert tubes*," like those of glass or lead in the body, and no way concerned in the circulation, except as conduits from the heart to the veins! Yet such has been the cry of most of those who have alluded to Dr. Parry's writings.

We shall now proceed to a short exposition of what we believe to be as nearly the true state of the circulation, in health, as observation and experiment have yet enabled us to ascertain. In this exposition we shall differ, in some particulars, from most, if not all our predecessors; but we hope to reconcile many discrepancies of opinion, and to shew that several doctrines which have apparently been based on unequivocal experiments, are, in reality, but mere illusions.

We shall commence with the left ventricle of the heart in a state of distension, leaving the cause of that distension, for the present, out of consideration. The blood is projected by the ventricle into the aorta, already in a state of distension itself. The blood being an incompressible fluid, or nearly so, we admit, with Dr. Parry and others, that every column, in the arterial system, feels the shock or force of the blood's momentum, at the same instant. This we would be led to expect from reasoning and from the laws of hydraulics; but it is proved by the sight and touch—by the sight in vivisections, and by the finger in the living body, for the systole of the ventricle and the pulsations of the arteries, are precisely simultaneous in every part of the system, and wherever we apply our finger—whether that be on the carotid, the radial, or the posterior tibial. Now this could not possibly be the case, if only that portion of the arterial system nearest the heart—say the aorta, carotids, and subclavians, felt the increase of quantity occasioned by the discharge from the ventricle. The fact evidently is, that the whole arterial system feels the shock, and *equally* experiences the augmentation of blood, at the moment of its ejection from the ventricle. Now what is the quantity received at each stroke of the heart? About twelve fluid drachms—the ejection or systole occupying about one third of a second, and the diastole two thirds, or double that time, allowing the pulse to be at 60 in the minute. Supposing, then, that *no blood* was discharged from the arterial system during the reception of these twelve drachms, (which, however, is evidently not the case) we would be glad to ask those physiologists who argue for alternate dilatation and contraction of the arteries corresponding with the pulse, what perceptible or tangible increase of diameter in the vessels of the *whole arterial system* can be expected from such a quantity? We would urge this question still more forcibly, when we assert, as they will admit, that during this reception of the ventricular blood, the flow from the arterial to the venous system is, at least equal to what it was during the diastole of the heart; consequently, the whole arterial system does not contain, during the systole, an over-plus of twelve drachms, but only of about eight drachms of blood. Thus, while we freely admit, that the arterial system does actually contain an augmented quantity of blood during each systole, and, consequently, that there is an augmented diameter of the vessels, we deny that this increase of quantity or diameter can possibly be perceptible by the eye, or appreciable by the minutest or most accurate admeasurements. We now see the reason why neither Parry, nor Haller, nor Bichat, nor Hunter himself, could discover any dilatation or contraction in a denuded artery—for, in truth, these dilatations and contractions, to be made sensible to the eye, would require, not twelve drachms, but some pounds of blood, to be injected into so extensive a series of tubes, as that composing the arterial system of a large animal.

Although we think, that enough has been said to convince our readers, that no *perceptible* increase of diameter obtains in arteries during the systole of the ventricle, yet, we shall glance slightly at the statements of a few of the most eminent men who have contended for the said dilatations and contractions as the cause of the pulse. Spalanzani, Haller, and Senac, are the champions particularly quoted by the party who support the present doctrine of the schools.*

Let it be borne in mind that Spalanzani's experiments are chiefly microscopical (the most fallacious of all experiments) and made on cold-blooded animals, as salamanders, lizards, frogs, &c. In these, and some other animals of similar conformation, the aortæ are often a kind of appendage to the heart, and will dilate and contract for days after they are removed from the body—a circumstance which is never seen in the arteries of warm blooded animals. It is on these very varieties of structure in the two classes of animals, that Haller has founded his well-known argument against the influence of the arterial system in the circulation of man and warm-blooded animals. And, after all, Spalanzani does not draw the unqualified inference respecting the pulse, as dependent on dilatation and contraction of the artery, with which he has been charged. He believed that the heart was the sole agent in the circulation, and that its impulse extended even to the veins—that the motion of the blood could take place without any dilatation or pulsation—and, finally, that the successive motion of pulsation, though he thinks it does take place, cannot be perceived by the eye.

Haller had great doubts as to alternate dilatation and contraction of arteries being the cause of the pulse. He never could discover, and did not admit, these alternate states in cold-blooded animals—he never could, by chemical or mechanical stimuli, produce contraction in the aorta of any ani-

* We shall here be obliged to recapitulate a few passages printed in our first, or *Quarterly Series*, vol. II. p. 205, *et seq*. in order not to leave the train of arguments defective. This repetition, however, is of less consequence, as the former series is not now in the hands of one half of our present readers.

mal, or, without difficulty, in the other arteries of warm-blooded animals—in most cases, he could perceive no contraction—in all, so trifling an appearance of this kind, as to render it almost a questionable condition—he maintained the sufficiency of the heart to carry on the circulation—believed that the arterial dilatation and contraction impeded the progress of the blood—considered elongation of the vessel as one cause of the pulse—and admitted the existence of pulsation, where no alternate movement in the artery had taken place.* In respect to the *muscular* contractility of arteries, so far was Haller from drawing the unqualified conclusions of some moderns, that, in his last publication, in 1777, he speaks of this power in arteries as—"debilem, quam neque sponte agentem unquam viderim, neque irritatio mechanica suscitet."

It is Senac, however, who has laboured most to prove the alternate dilatation and contraction of arteries in correspondence with the pulse. Yet, he is able to offer no proof but the supposed necessity of the case; and, indeed, he admits that the circumference of arteries does not extend so much as their axis; while, with regard to any positive evidence as to the states of alternate dilatation and contraction, he confesses that no such circumstance takes place in the vessels of cold-blooded animals, which may be compared, in his opinion, to tubes of glass. He acknowledges, further, that the dilatations in the *successive portions* of the arterial tube cannot be seen even in man "Les yeux ne peuvent pas saisir cette suite successive de la dilatation dans les arteres." We are astonished, indeed, how the clear mind of Senac should be so warped by a favourite hypothesis, as to imagine that there could be a *successive* dilatation through the arterial system, when his own hands might have ascertained, that the pulsations are perfectly *synchronous* from the root of the aorta to the minutest plantar twig. Or could he suppose that a *wave* of blood would flow, or rather *fly*, from the heart to the sole of the foot, like a ray of light, without requiring any appreciable time for this march? Finally, we may remark, that Leeuwenhoek, the prince of microscopical observers, never saw the dilatation and contraction of arteries in the course of

---

* If we put our hand on any leaden water-pipe where a pump is acting at one end, we shall feel a pulse at each stroke of the piston, precisely resembling that which we feel in touching an artery in the living body. In the intervals between these pulses, the tube, in the same manner as the artery, conveys no particular sensation. It is, in fact, the *rush* of water through the one, and blood through the other, that causes the phenomenon of the pulse—and not any dilatation or contraction in either of the tubes here mentioned.—*Ed.*

his multifarious experiments on the circulation of different animals.

Bichat, the modern Haller, has taken great pains to shew, that the arteries do *not* exhibit this alternate motion of contraction and dilatation. He repeatedly laid bare the carotid artery, and always found that the whole tube was agitated by a kind of locomotion—" on voit evidemment que tout le tube est agité d'un mouvement de loccomotion commun." And again, he says, " dans les battemens des arteres les vaisseaux sont, pour ainsi dire, passifs." He asserts, that dilatation and contraction in the arteries (corresponding with the pulse) must be next to nothing, since it is inappreciable by the sight. " La dilatation arterielle, et par consequent la contraction, sont presque nulles, ainsi ne peut-on point les appercevoir."

We shall only allude to one or two other authorities on the present subject.

It is well known that John Hunter was very undecided in respect to arterial dilatation and the nature of the pulse. He asserts that, the best evidence, actual observation, cannot be trusted, as to any alternate movement in the arteries, and believes that the diastole of the arteries consists much more in an elongation of their tube, than in its dilatation; and, therefore, that the term diastole would be more properly used to express the elongated state.

Mr. Charles Bell, in his system of dissections, conceives that the pulsation in the arteries is occasioned by the *whole quantity* of blood sent through them in the direction of their axis, lengthening them in opposition to their elasticity, and causing them to form contortions or curves. " It is not (says he) the degree of *dilatation* which we feel in the pulse, but the shock given to the column of blood by the action of the ventricle."

The accurate and philosophical Dr. Young, after examining the various powers concerned in the circulation, concludes that the pulse, as perceptible to the touch, must obviously depend entirely on the action of the heart, since the state of the arteries can produce little, if any, effect on it.

We have gone farther into the subject of *perceptible* dilatation and contraction of arteries in correspondence with the pulse, in order to shew the rising generation of students how complacently doctrines are delivered as gospel in the schools, when there really is not any proper proof in their support, and when proofs innumerable may be brought against them, furnished, even, by the party who maintain the doctrines in question.

The fact of non-dilatation of the *trunks* of arteries, *in any perceptible degree*, at each systole of the ventricles, inevitably brings with it this consequence, that the flow of blood cannot go on in those trunks, *in any perceptible degree*, during the diastole of the ventricle. We would ask those who are shocked at the idea of the blood being alternately quiescent and in motion in the *trunks* of arteries, how it is possible for the current to proceed, say in the aorta, while the ventricle is dilating, while the semi-lunar valves are shut, (for if they are not placed there for the purpose of shutting during one state, and opening during the other state of the ventricle, they must be useless) when there is no blood pouring into the aorta, and when the aorta itself is not perceptibly contracting? We unhesitatingly assert, that if the blood continues to move on during the above state of things, then it must leave an absolute and complete vacuum at the root of the aorta, which has never been found any where else on the globe we inhabit. The supporters of perpetual motion cannot deny this inference; but they offer in opposition what they call matters of fact. Look, say they, with your microscope, at the capillaries of an animal, and you will see nothing but a current of blood, like a rapid river pursuing a uniform course. Or, say they again, cut the *trunk* of an artery, and the flow will be *perpetual* though not uniform. In respect to *capillary* circulation, we readily grant, that the motion of the blood is constant there, though by no means so rapid as it is represented. We grant, that whatever quantity of blood is accumulated in the arterial system during ventricular contraction, (say two thirds of the whole, or eight drachms) must pass through the capillaries during ventricular dilatation, and, thus, a constant stream is kept up. But this quantity is not sufficient to produce any evident dilatation of the trunks of arteries, and, consequently, the flow through *them* must be as inevident as the augmentation of size. And who, we ask, has seen the motion of the blood in the trunks of arteries? Have the eyes of experimenters, even with their microscopes, been able to see through three coats of an artery? We rather think not.* The only approach to such a thing is to be found in Bichat, and let us hear what he says. "We may derive, says he, a very exact idea of the circulation by examining the *mesenteric arteries* of a living animal through the peritoneum. At *each pulsation* they appear to be *raised at once from their origin to their extremities*. The motion is *not*

* "In the larger arteries (says Dr. Thomson) of the mesentery, or of the web in the foot of a frog, *the globules of blood are never to be seen in ordinary and healthy circulation*."—"Our being able to distinguish the globules, *even in the capillary vessels*, is at all times the consequence of *diminished circulation*." P. 81.

*progressive;* and the blood does *not flow,* properly speaking, but is *pushed suddenly* by a gentle *shock.*"—*Anatomie Generale.* The reader will find the passage thus translated in Rees's Cyclopedia, article "Heart," and, we believe, by Mr. Lawrence. We leave the advocates of perpetual and uniform motion to reconcile this statement of the most able experimenter of the age with their doctrines; while we appeal to the unprejudiced reader, whether this be not as direct and unequivocal a proof of the doctrine we maintain, respecting the alternate quiescence and flow of blood in the *trunks* of arteries, as any vivisection can possibly afford. The illustrious physiologist just quoted, goes on to say that, "as the arteries are always full of blood, the impulse produced by the left ventricle, is felt *at once* through the *whole system* to its very extremities." "If a tube, says he, dividing into a series of branches, which were again divided for any number of successive times, and completely filled with fluid, were connected to a syringe, the depression of the piston would cause an efflux from all the open branches *at that very instant.*" He considers, in fact, and very justly too, that ventricular contraction, general movement of the whole mass of arterial blood, and passage of this blood into the capillary system, are three circumstances that occur *at the same moment.* "Hence, says he, each ventricular ejection of blood remains some time in the arteries before it reaches the capillary system." These statements of an accurate anatomist who had no hypothesis in view, harmonize rather curiously with the doctrine of rapid and uniform motion of the blood.

Again, if we denude an artery, and press it between the finger and thumb, we feel the pulse or rush of blood through it very distinctly at each systole of the heart; but, during the diastole of the same organ, the parietes of the artery, even although we bring them in actual contact, feel just like so much of a dead tube, without communicating one particle of a sensation similar to that which they, the moment before, communicated. Now, if the motion of the blood through the artery be only *accelerated* during the systole of the ventricle, and still more if it be a uniform rapid river, as the microscopic philosophers represent it, how comes it to pass, that, during the diastole of the ventricle, it exhibits *no degree* of the same phenomena manifested during the systole? As far, then, as *tangible* evidence goes, in respect to the blood's motion in the trunks of arteries, (and we deny that the experimenters have any other *ocular* evidence besides what Bichat has also had) it is completely in favour of alternate motion and quiescence. When we say *quiescence*, be it always understood, that we mean such a degree of quiescence as renders the little progressive motion it may have, imperceptible to the eye, or inappreciable by other means hitherto devised.

The argument found on the flow of blood from a wounded artery is almost too futile to need refutation, were it not still brought forward by some late writers of note. We have before* pointed out how very erroneous a notion we form of the healthy circulation by the flow of blood from a wounded artery. When a large vessel is cut, the whole blood of the body rushes to that point—qua data porta—and consequently more blood passes along the cut artery in a minute than would naturally pass in half an hour. A pipe bursts in our streets, and the water boils out with great force and velocity, even if the fluid be perfectly quiescent the moment before. The pressure of the reservoir at a distance has precisely the same effect, in such a case, on the motion of the water, as the action of the heart and the contractile power of the arteries have on the blood, when a wound is made in the vessel. In both instances the velocity of the fluids through the artificial outlets, as compared with the previous motion in the tubes, is great beyond comparison. Bichat, indeed, has very justly remarked that—"the progress of physiology concerning the circulation has been much obstructed by the current notions concerning the *velocity* of the blood's motion in the arteries, of which we can form no true estimate, because the motion is not progressive, and cannot be subjected to calculation."

And this leads us to notice a source of fallacy in microscopic observations on the circulation of the blood, which we are surprized has not struck the experimenters themselves —it is the circumstance of the field of vision being magnified by the glasses used. Is it not obvious, that if a piece of the membrane of a frog's foot, say one-eighth of an inch in breadth, be magnified by glasses to the appearance of four inches, the velocity of a globule of blood passing across this space is also magnified to 32 times its real rate? Let any one, for example, take a telescope and look through it at a boat on the Thames, while he stands on the bank. It will appear to fly through the water. Let him reverse the telescope—it will appear almost a fixture; though the boat is moving all the while at an equal rate of progression. Here then is an explanation of the "*rapid rivers*" which the microscopic philosophers have discovered in the circulation—too forcibly reminding us of the gigantic animal which was seen to stride across the moon, a thousand miles at a step, when an insect had got between the glasses! If to this tre-

* Vol. II. *Quarterly* Series, October 1819, p. 211.

mendous source of fallacy in the microscope, we add the panic, the pain, the convulsions, and the contortions of an animal on the rack of experiment, we shall have some clue to the contradictory statements of physiologists—scarcely any two of whom agree on any one circumstance regarding the circulation! Some of them see nothing but "rapid rivers"—others observe currents and counter-currents "hurrying too and fro"—some see the ventricular impulse distinctly, not only in the branches and capillaries of the arteries, but even in the *veins*—nay, Dr. Thomsom saw the alternate impulse more distinctly in the *veins* than in the arteries!! "They can be traced," says he, "into the veins; and, indeed, in most of my experiments, *these accelerations* from the pulse of the heart were more visible in the veins than in their corresponding arteries." What are we to conclude from this chaos of microscopic contradictions, or, as Dr. Charles Parry has aptly designated them—"*opposite* matters of fact?" Why, that they are all illusions—the more dangerous because they wear the garb of realities. It is far safer to argue from no data at all, than from false data.

But can we glean no help from common observation of the phenomena presented during health or disease, without the aid of lenses? Nature has very often converted the arteries of the living body into the ridiculed "inert tubes," about which so much has been said, and where, consequently, there could be no dilatation and contraction at all, corresponding with the action of the heart. What was the effect of this dreadful state? Why, that some of them lived to the age of 130 years. Old Bayles, recorded in the Philosophical Transactions, attained this age, almost the whole of his arteries being bony or cartilaginous. Now, we would ask whether it is likely that there was a constant stream through these inert tubes, whether the ventricle was delivering blood into them or swelling out and filling itself? If the blood was not alternately quiescent and flowing in these vessels, then there is a law for them which exists no where else in heaven or on earth, that we know of. "Oh!" but says the experimenter, "in these states of ossified arteries there are faintings, palpitations, and other phenomena resulting from the unnatural condition of the vessels." Are there indeed? No mention is made of faintings in the case of Old Bayles. He had intermissions of the pulse it is true; but will any physiologist of the present day attribute that phenomenon to an ossified artery? Is it not clearly attributable to disease of the heart, or rather of the *valves?* "Ossea," says Haller, "arteriarum natura *frequens* omnino senum vitium est. Et tamen senes iique homines in quibus arteriæ a morte osseæ visæ sunt, diu cum ejusmodi rigidis et ab omni contractione alienis arteriis vixerunt. Neque enim hoc vitium subito opprimit." Morgagni (Let. xxiii.) found the aorta and iliacs converted into bone. The woman had palpitation; but Morgagni by no means attributes it to the ossification, for he says, "at the same time, it is necessary to declare why, *out of so great a number* of persons in whom there was an aorta *of this kind*, many of them certainly did *not* labour under these disorders at all." Fallopius asserts "that he had seen *all the arteries* in the left half of an old woman degenerated into BONE." Instances, indeed, of this kind are innumerable, and they are calculated to carry a strong degree of conviction to the unprejudiced mind. They prove that the heart alone is capable of carrying on the circulation as far as the capillaries; and what may take place *there* is nearly to us unknown. We cannot help thinking, however, that the important functions of secretion, nutrition, absorption, &c. are enough for the capillaries, without giving them the additional drudgery of *impelling* the blood, for which there is the strongest muscle in the body especially appointed, aided, we allow, by the tonic and elastic power of the arteries.

Our ideas of the circulation are finally these. The *ventricular contraction* throws a small quantity, say an ounce and a half, of blood into the aorta, which gives an impulse to every column of blood in the whole body, and augments the whole *arterial* system *equally*, and consequently in a degree totally inappreciable by the eye or any other means. During the *diastole* of the ventricle, the tonic and elastic powers of the arteries cause a discharge from the whole arterial system of the overplus accumulated during the systole; which discharge is also so small as to produce no *evident* diminution of the arterial calibres, and consequently no *appreciable* current along their trunks, though there must, of course, be a flow (somewhat diminished we think) through their capillary terminations. This process may be rudely illustrated by the action of a pair of double or smith's bellows. The action of the *lower bellow* augments the blast through the pipe, and throws some air through the valve into the upper bellow, which serves as a reservoir. When the lower bellow is dilating and filling, the upper bellow continues a diminished stream of air through the pipe, expending the overplus it had received during the action of the great bellow below—and so on. Now if the upper bellow, which may represent the arterial system, was so large that the portion of air forced into it did not *ostensibly* increase its dimensions, the parallel would be nearly complete—for then the air in the upper bellow would have so little motion as to be also imperceptible, though the current would be constant (but unequal) through the pipe.

It appears to us (and we believe that little more than conjecture can yet be offered on the occasion) that the *vis a tergo* is perfectly equal to the passage of the blood through the capillary system. We see this passage forced or effected by the arteries alone after death,* and consequently after the heart's action has ceased. And we see no reason why the heart and arteries together should not be able, during life, to accomplish what the arteries unaided perform immediately after life has ceased. Considering too that the blood is an incompressible fluid, we see no just cause why the same *vis a tergo* should not propel it along the channels of the veins. These channels being much more capacious than the arteries may cause the flow of blood to be more placid and uniform in the veins than in the arteries—though we have no unequivocal proof of the velocity or uniformity of the venous current, microscopic observations being discordant, and the coats of the venous trunks offering insuperable bars to ocular evidence. Every man must have seen the blood flow, *per saltum*, from a vein in the arm, and it is quite absurd to attribute this phenomenon to the beating of the subjacent arteries, when we now know that they have no perceptible dilatation or contraction. We must be convinced that the blood is received *periodically* from the *head* of the venous column by the heart, and it is extremely difficult to conceive how this periodicity should fail to affect the motion of an incompressible fluid in the great trunks leading to the central organ. Indeed all experimenters have observed a reflux of blood, and bulging out of the head of the cava, on each contraction of the heart. Independently of the *vis a tergo*, however, there can be little or no doubt that the heart has a capability of actively dilating as well as con-

---

* Dr. Philip, for instance, having passed a ligature round the great cardiac vessels of a frog, and then cut out the heart, found that the *circulation* continued in the capillaries for some time, and then gradually ceased. This indeed is an old experiment, and has been perspicuously explained both by Hunter and Parry. The latter gentleman has clearly shewn that the larger arteries continuing to contract by their tonic and elastic properties, after the heart has ceased to supply them with blood, they necessarily propel all, or nearly all, the blood they contain, through the capillaries into the veins. When their tonic or fibrous power dies, the elastic power endeavours to bring back their diameter to a certain width, but not being *able* to suck the blood from the veins we find the arteries *flattened* in the dead body, and empty of course. In ossified arteries, on the contrary, we find the blood still in them after death. How can it, in fact, leave them, without leaving, as we before remarked, a vacuum in the aorta? for in this ossified state they cannot contract upon it.

tracting. This is proved by hearts recently cut out of animals. If they be put into warm water they will continue to dilate and contract, for some time, thus sucking in and squirting out the water from their cavities. And thus it is, that if a ligature is put round the arm of a man whose veins are very prominent, we see those below the ligature swell, and those above it shrink—evincing the *vis a tergo* below the obstruction, and the suction of the heart above it. When we contemplate the structure of the auricle and ventricle, it appears quite impossible that the contraction of the former can be destined for the dilatation of the latter. The active dilatation of the ventricle, by rapidly sucking the blood from the auricle, will cause it to fall in, of course, and appear to be contracting and filling the ventricle; but it really seems to be little more than a reservoir for the master-agent the ventricle. When the ventricle contracts, it stops, of course, the rush of blood through the auricle and cava, and then the fluid accumulates in the auricle or reservoir for the next expansion of the ventricle.

We believe the above exposition to be nearly the real state of the case; but it must be confessed that it is exceedingly difficult, if not impossible, to ascertain, by unequivocal experiments, the functions of the complicated machinery employed in the circulation of the blood. We must therefore trust partly to reasoning and reflection, and partly to observation.

We see then that the heart is placed between the venous and arterial systems, and appears to be the great agent that regulates the equilibrium of the blood throughout both. If it act too forcibly, it distends the arterial system beyond the medium, and these vessels, by the reaction of their elastic and tonic powers, must distend the capillaries also, and accelerate the circulation through them. If, on the other hand, the action of the heart be below par, and does not keep the arterial system at the due pitch of distension, it is evident the arteries must, by their own powers go on lessening their diameters, while the balance of blood is thus thrown on the venous system, which is not emptied in due time and degree by the heart. The former state must be that of arterial distension—the latter that of venous congestion. Great vacillations, however, of this kind, may take place in a sound constitution, and perfectly consistent with health. The common muscular motions which we are hourly calling into action in the avocations of life, are hourly disturbing this harmonious balance of the circulation, without inconvenience. Muscular motion certainly accelerates the *venous* circulation, as is evident from the familiar example of grasping a stick when the blood is flowing from a vein; but by hurrying the blood to the heart, it appears to excite the central organ also to quicker action, and then, of course, the whole circle of the circulation is accelerated. The causes indeed which disturb the balance of the circulation are very numerous. Every atmospherical change of temperature increases the quantity of blood in one set of vessels, and lessens it in another. The cold and warm baths offer striking examples which every one can appreciate. Even the passions have the power of perturbing the circulation as well as the temper. The effects of violent emotions, as rage or terror, are familiar to all observers; but, in truth, there is not a single emotion of the mind, whether of joy or sadness, admiration or aversion, hope or fear, love or hate, solitude or confidence, that does not, through the instrumentality of the nerves, disturb the even balance of the circulation. And wisely has the Divine Architect provided against these constant occurrences in life. Every vessel and set of vessels throughout the whole machine are endued with a surprising power of dilatation and contraction (whether active or passive,) or in other words, of accommodation to the ever-varying magnitude of the stream passing along their channels. These are most important properties—not only warding off innumerable evils, but correcting them when they actually occur. It is principally for these wise purposes, we imagine, that the vessels were endued with the properties in question; though physiologists have too exclusively viewed them as powers mainly instrumental in the common course of the circulation. This common course, we verily believe, is *chiefly* carried on by the heart, a powerful organ most admirably fitted for its office. At the same time, we have admitted the auxiliary assistance of the arteries in the manner so fully descanted on.

As we have confined this article entirely to the *physiology* of the circulation, we have taken very little notice of Dr. Lucas's observations on the subject. They very nearly coincide with our own, excepting as to the degree of quiescence in the trunks of arteries during ventricular relaxation. On this point we expect few converts from a doctrine taken in with the first rudiments of anatomical education, and firmly rooted in the mind. We leave our observations for the consideration of those who are commencing their studies, and who may perchance examine for themselves as they proceed. The main part of Dr. Lucas's work—the pathology of the vascular system, will come under review in our next number. In the mean time we may take leave to say that his work is characterized by much clearness of perception, and contains as luminous an explication of the intricate subjects discussed as we can yet expect from the imperfect data on which our doctrines are founded.

